# Population structure of the dengue viruses, Aragua, Venezuela, 2006–2007. Insights into dengue evolution under hyperendemic transmission

**DOI:** 10.1016/j.meegid.2011.12.005

**Published:** 2012-03

**Authors:** Rosmari Rodriguez-Roche, Elci Villegas, Shelley Cook, Pauline A.W. Poh Kim, Yoandri Hinojosa, Delfina Rosario, Iris Villalobos, Herminia Bendezu, Martin L. Hibberd, Maria G. Guzman

**Affiliations:** a “Pedro Kouri” Tropical Medicine Institute, P.O. Box 601, Marianao 13, Havana, Cuba; b Instituto Experimental “José Witremundo Torrealba”, ULA, Trujillo, Venezuela; c Natural History Museum, Cromwell Road, London SW7 5BD, United Kingdom; d Genome Institute of Singapore, Singapore 138672, Singapore; e Hospital Central de Maracay, Universidad de Carabobo, Venezuela

**Keywords:** Dengue, Evolution, Phylogeny, Venezuela

## Abstract

During the past three decades there has been a notable increase in dengue disease severity in Venezuela. Nevertheless, the population structure of the viruses being transmitted in this country is not well understood. Here, we present a molecular epidemiological study on dengue viruses (DENV) circulating in Aragua State, Venezuela during 2006–2007. Twenty-one DENV full-length genomes representing all of the four serotypes were amplified and sequenced directly from the serum samples. Notably, only DENV-2 was associated with severe disease. Phylogenetic trees constructed using Bayesian methods indicated that only one genotype was circulating for each serotype. However, extensive viral genetic diversity was found in DENV isolated from the same area during the same period, indicating significant in situ evolution since the introduction of these genotypes. Collectively, the results suggest that the non-structural (NS) proteins may play an important role in DENV evolution, particularly NS1, NS2A and NS4B proteins. The phylogenetic data provide evidence to suggest that multiple introductions of DENV have occurred from the Latin American region into Venezuela and vice versa. The implications of the significant viral genetic diversity generated during hyperendemic transmission, particularly in NS protein are discussed and considered in the context of future development and use of human monoclonal antibodies as antivirals and tetravalent vaccines.

## Highlights

► We examine the population structure of dengue viruses in Venezuela, 2006–2007. ► Phylogenetic trees show that only one genotype was circulating for each serotype. ► Extensive viral genetic diversity indicates significant in situ evolution. ► Particularly, variations in non-structural proteins appear to have a major role. ► These results on dengue evolution are relevant in the context of vaccine design.

## 1 Introduction

DENV is a single-stranded, positive-sense RNA virus belonging to the genus *Flavivirus*, family *Flaviviridae*. There are four antigenically distinct serotypes (DENV-1 to -4), all of which can cause illness. Dengue has a wide spectrum of clinical presentations, often with unpredictable clinical evolution and outcome. While most patients recover following a self-limiting non-severe clinical course, a small proportion progress to severe disease, mostly characterised by plasma leakage with or without haemorrhage (WHO/TDR, 2009). In many tropical and subtropical countries DENV virus constitutes a major public health problem. During the past 30 years or so, in the Americas, dengue disease has increased dramatically. Over 4.5 million cases were reported during 2000–2007, compared with approximately 1 million cases previously reported in the 1980s. Likewise, the number of DHF cases increased over time from 13,398 (0.2/100,000) during the 1980s, to 111,724 (1.7/100,000) during 2000–2007. From 1980 to 2007, Brazil reported the majority of dengue cases (54.5%). However, Venezuela reported the highest number of DHF cases (35.1%) during the same period (San Martin et al., 2010).

The first dengue epidemic was reported in Venezuela in 1964, which lasted until 1967 (PAHO, 1979). During this time, 23 deaths were attributed to DENV, but no investigations were carried out to confirm the occurrence of DHF/DSS as the cause of death. Afterwards, an outbreak due to DENV-2 was reported in 1969. Subsequently from 1971 until 1977, the small number of reported indicated that DENV activity was low (PAHO, 1979). However, DENV-1 was introduced in 1977, firstly into Jamaica and Cuba and 1 year later, in Venezuela and Puerto Rico causing massive epidemics of dengue (PAHO, 1979). During the following 4 years, DENV-1 spread throughout the Caribbean Islands, Mexico, Texas, Central America and South America. In 1981, DENV-4 was introduced into the Americas but did not cause a major epidemic in Venezuela. Also in 1981, a DENV-2 strain of Asian origin was introduced into Cuba, causing the first epidemic of DHF in the Americas (Guzman et al., 1995, Kouri et al., 1986, Kouri et al., 1987). However, a second large epidemic of DHF occurred in Venezuela in 1989–1990 (PAHO, 1990). At the same time DENV-1, DENV-2 and DENV-4 were circulating although DENV-2 virus was associated with most fatal cases (Gubler and Meltzer, 1999). DENV-3 re-appeared in the Latin American region in 1994 after an absence of 17 years (Guzman, 1995). The virus was detected almost simultaneously in Panama, causing a small outbreak of classic DF, and in Nicaragua, where it was associated with a nationwide epidemic of DF/DHF. After the spread of the virus to the Latin American region this serotype re-appeared in Venezuela in 2000 causing the largest dengue epidemic in Venezuela since 1989 (Uzcategui et al., 2003). Currently, like in most Latin American countries, dengue is endemic in Venezuela but the population structure of the viruses being transmitted is not well understood. Phylogenetics has enhanced our understanding of DENV population dynamics and sizes at various stages of infection and transmission and this information actually improves our ability to predict DENV emergence (Weaver and Vasilakis, 2009). Therefore, the present investigation was aimed at characterisation of the DENV serotypes and genotypes circulating in Aragua State, Venezuela for the purposes of understanding viral epidemiology and to provide useful information for the potential development and use of antivirals and vaccines.

## 2 Material and methods

### 2.1 Patient enrolment and blood sample collection

Blood samples from suspected dengue cases were collected at the Maracay Central Hospital, Venezuela, during the period November 2006–April 2007 as part of the DENCO project: Towards successful dengue prevention and control (Alexander et al., 2011). Written informed consent was obtained from all patients or guardians. The study received approval from the Ethics Committee of Experimental Institute “José Witremundo Torrealba”, Ethics Committee of “Pedro Kouri” Tropical Medicine Institute and from the World Health Organization Research Ethics Review Committee (number A70175).

### 2.2 Diagnostic tests

After separation from red blood cells, acute-phase serum samples were tested for DENV RNA using a nested reverse transcriptase–polymerase chain reaction (RT-PCR) assay that targets the capsid-premembrane region and allows both DENV detection and serotyping (Lanciotti et al., 1992). Dengue positive samples were studied using real-time RT-PCR to measure viral titre using a serotype-specific assay that has been described previously (Laue et al., 1999). Viral isolation was also attempted by inoculation onto *Aedes albopictus* C6/36 cells (Rodriguez-Roche et al., 2000). Serotyping after viral isolation was performed by indirect immunofluorescence using monoclonal antibodies (Henchal et al., 1983).

### 2.3 Full-length viral genome amplification

Real-time PCR and/or viral isolation positive samples were processed in order to amplify. The earliest acute sample of each patient enrolled in the study was utilised for sequencing purpose. Briefly, viral RNA was extracted from 140 μL of serum sample using the QIAamp viral RNA mini kit (Qiagen, Germany). cDNA was synthesized using the SuperScript III First-Strand Synthesis System (Invitrogen, USA) using specific serotype primers complementary to the 3′ UTR as described previously (Christenbury et al., 2010). An aliquot of 3 μl cDNA was subjected to PCR using the Expand High Fidelity PCR System (Roche Applied Science, Germany) according to the manufacturer’s instructions. Five pairs of primers for each serotype were utilised, designed to obtain five overlapped fragments (F1–F5) covering the complete genome of the viruses (Christenbury et al., 2010).

### 2.4 Nucleotide sequencing

PCR products were purified using the QIAquick PCR Purification Kit (Qiagen, USA). Direct sequencing of these products was carried out using an Applied Biosystems BigDye ddNTP capillary sequencer as described previously (Schreiber et al., 2009). The chromatograms from capillary sequencing were assembled into a specimen consensus sequence using SeqScape version 2.5 (Applied Biosystems). The minimum fold-coverage of the sequences was at least 2×, but in average it was 3×. The nucleotide sequences reported in this study are available in GenBank ID: HQ332170–HQ332190.

### 2.5 Sequence analysis

Full polyprotein nucleotide sequences of each dengue serotype obtained in the present study were aligned using ClustalX (Thompson et al., 1997) together with relevant sequences retrieved from GenBank (available from the authors on request) such that representative sequences from all the known DENV genotypes were present. From the initial data set, identical sequences and known recombinant sequences (as published by the authors) were removed from the alignments. This produced a total data set of 101 sequences for DENV-1 10176 nucleotides in length, 89 sequences for DENV-2 10173 nucleotides in length, 95 sequences for DENV-3 10170 nucleotides in length and 77 sequences for DENV-4 10161 nucleotides in length. Maximum likelihood (ML) phylogenetic trees were estimated using the general time-reversible model (GTR) of nucleotide substitution, with the GTR substitution matrix, the base composition, the gamma distribution of among-site rate variation, and the proportion of invariant sites all estimated from the data using Modeltest (Posada and Crandall, 1998). To assess the robustness of particular phylogenetic groupings, a bootstrap analysis was undertaken using 1000 replicate neighbour-joining trees using the ML substitution matrix described above. All analyses were performed using PAUP (Swofford, 2003).

Phylogenetic analyses were also performed using Bayesian analysis in MrBayes v3.1.2 (Huelsenbeck and Ronquist, 2001), with a minimum of 20 million generations and a burn-in of 10%. Stationary was assessed at effective sample size (ESS > 400) using Tracer v1.4.1 (part of the BEAST package) (Drummond and Rambaut, 2007).

All Bioinformatic analyses were carried out on the freely available Bioportal: www.bioportal.uio.no.

## 3 Results

From 50 acute-phase sera, 31 samples yielded viral isolates and/or were positive by RT-PCR tests (10 DENV-1, 10 DENV-2, 2 DENV-3 and 9 DENV-4), confirming the co-circulation of the four DENV serotypes in Aragua State, Venezuela. All positive samples were subjected to Reverse Transcription and PCR for full-length genome amplification using the long PCR system mentioned above. Twenty-one of 31 DENV were fully amplified directly from sera (7 DENV-1, 7 DENV-2, 2 DENV-3 and 5 DENV-4) (Table 1). The viral titre determined for each serum sample had a significant influence on the likelihood of attaining a genome length sequence. Real time RT-PCR positive samples ranking from 12,880 to 8 PFU/mL produced positive long PCR products useful for sequencing. However, 10 samples with less than 4 PFU/mL resulted in unsuccessful long PCR amplification. The low viral titre observed in these patients could be due to the time of sample collection >3 days following fever onset, presumably in these cases viral clearance through an adequate immune response to the infection was responsible for the mild disease observed amongst this group.

**Table 1:** Data corresponding to 21 Venezuelan samples sequenced in the study.

Sample number	Age (years)	Date of fever onset	Days after fever onset	Serotype	Clinical classification^a^	Type of infection^b^	Viral titre (PFU^c^/mL)
61006-1	19	05/11/2006	3	DENV-1	Non-severe	S	57.5
61059-1	7	29/11/2006	3	DENV-1	Non-severe	S	39.1
61060-1	4	30/11/2006	2	DENV-1	Non-severe	S	8994
61063-1	3	02/12/2006	2	DENV-1	Non-severe	P	25.9
61068-1	7	09/12/2006	3	DENV-1	Non-severe	S	357.7
61081-1	11	20/01/2007	3	DENV-1	Non-severe	P	18.3
61084-1	9	22/01/2007	3	DENV-1	Non-severe	S	273.7
61069-1	38	10/12/2006	3	DENV-2	Non-severe	S	35.6
61082-1	27	20/01/2007	4	DENV-2	Non-severe	S	1578
61095-1	<1	08/02/2007	4	DENV-2	Severe	P	6082
61115-1	21	17/03/2007	2	DENV-2	Severe	S	942.7
61133-1	52	04/04/2007	3	DENV-2	Severe	S	2277
61136-1	5	08/04/2007	1	DENV-2	Non-severe	S	12890
61154-1	15	27/04/2007	3	DENV-2	Non-severe	S	33.47
61035-1	51	20/11/2006	2	DENV-3	Non-severe	S	280
61051-1	15	25/11/2006	3	DENV-3	Non-severe	S	212.3
61013-1	12	06/11/2006	4	DENV-4	Non-severe	S	8.04
61027-1	51	14/11/2006	2	DENV-4	Non-severe	S	4819
61054-1	12	25/11/2006	4	DENV-4	Non-severe	S	49.3
61073-1	29	02/01/2007	3	DENV-4	Non-severe	S	1035
61110-1	14	01/03/2007	2	DENV-4	Non-severe	S	5284

a Clinical classification is shown by the New WHO/TDR Guidelines published in 2009.

b S: secondary infection, P: primary infection.

c PFU: plaque formation units.

The analysis of the full-length viral sequences revealed the existence of a single genotype for each serotype in Aragua State during the period of study. However, the extent of genetic variability observed amongst viruses of the same genotype collected over a few months of study was surprising. The most variable serotype was DENV-1; although two isolates of this subgroup (VE_61059_2006 and VE_61060_2006), which were obtained from two members of the same family (with fever onset only one day apart), had identical sequences. Comparison with the five remaining DENV-1 revealed 2% of variable sites. On the other hand, comparison among the seven DENV-2 sequences and the five DENV-4 sequences revealed 1.3% of variable sites in both serotypes. No differences were found between the only two DENV-3 studied herein, which is most likely due to the limited number of DENV-3 isolates recovered.

Comparison of the unique DENV-3 sequence obtained in the present study with 12 full-length DENV-3 Venezuelan sequences available in Genbank, corresponding to isolates from the same locality (Aragua) and period (2006–2007) of study revealed up to 2.1% of divergence at the nucleotide and 1% at the amino acid level, respectively. A similar analysis was carried out for the other serotypes using full-length Venezuelan sequences available in Genbank, corresponding to isolates from the same locality and period i.e. 18 DENV-1, 8 DENV-2 and 22 DENV-4 sequences. Data for DENV-1 revealed up to 2.3% of divergence in nucleotide sequences and 0.9% at the amino acid level, 2.2% and 1% for DENV-2, 1% and 0.3% for DENV-4, respectively. Notably, DENV-4 showed lower variability despite the fact that a larger number of sequences were available for this serotype.

In general, for the four serotypes, most non-synonymous mutations produced conservative amino acid changes. Notably, these mutations were generally unique for particular isolates, suggesting that they were not fixed within the population during the time of study. However, significantly larger sample sizes will be required to verify this observation. Nonetheless, the comparison of Venezuelan DENV sequences obtained in this study and those corresponding to isolates from the same locality (Aragua) and period (2006–2007) available in Genbank with globally distributed DENV sequences, revealed specific amino acid changes in the Venezuelan isolates that appeared to be fixed after the introduction of particular genotypes into this country as well as specific amino acidic changes that differentiate Latin-American isolates from other geographically distant viruses but corresponding to the same genotype. Most of these changes occurred in the non-structural genes (Table 2).

**Table 2:** Summary of particular non-conserved amino acid replacements observed in recent Venezuelan isolates.

Serotype	Proteins				
	E	NS1	NS2A	NS4B	NS5
DENV-1		D146G^a^		H18Y^a^	
DENV-2		I93T^b^	A109T^b^	S24P^c^	
		T264I^c^	Q164L/R^a^^,^^b^		
DENV-3			K4E^a^		S229A^d^
					P365S^d^
					K371R^d^
					E374G^d^
					R389K^d^
					R422K^d^
					E429D^d^
DENV-4	A222T^a^	D290N^a^			
	S354A^a^				

a Amino acid replacements in Venezuelan isolates unusual for global sequences.

b Amino acid replacements in recent Venezuelan isolates compared with previous circulating lineages.

c Amino acid replacements in Venezuelan isolates from the same period (2006–2007).

d Amino acid replacements in Latin American isolates compared with others isolates corresponding to genotype III.

**Fig. 1:**
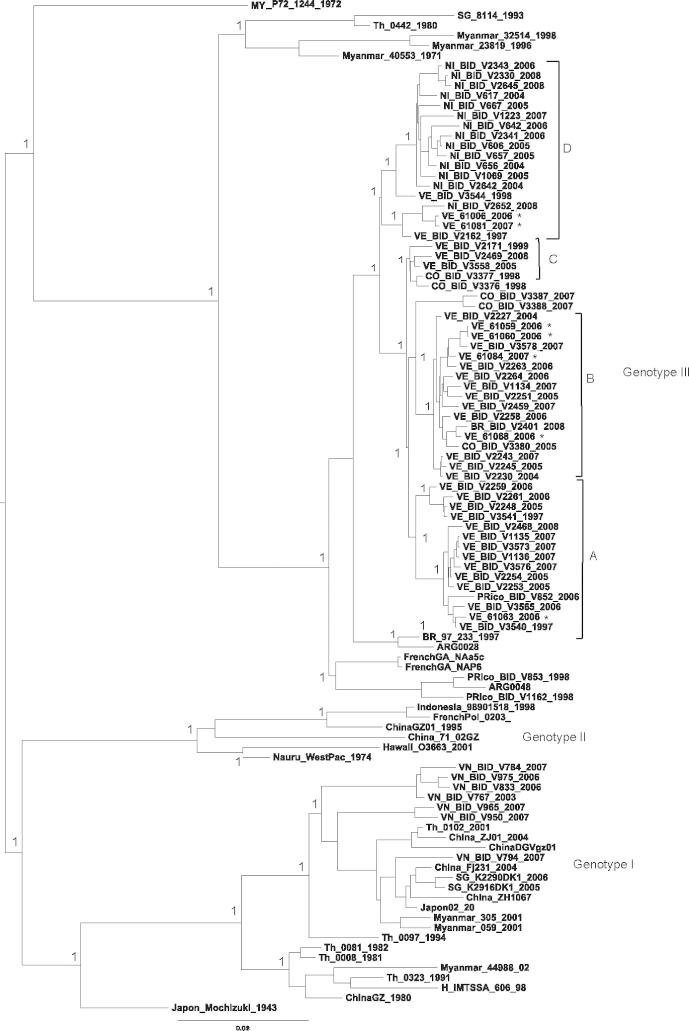
Bayesian phylogeny of DENV-1 polyprotein nucleotide data set, including Venezuelan isolates from 2006 to 2007. For clarity, posterior probabilities obtained via Bayesian methods are shown for the main clades only. Bootstrap support values obtained via 1000 neighbour-joining replicates were similarly high for these nodes. All horizontal branch lengths are drawn to scale; bar, 0.02 substitutions per site. The tree is midpoint-rooted for purposes of clarity only. The asterisks (∗) indicate those DENV sequences that are published for the first time in the current study.

**Fig. 2:**
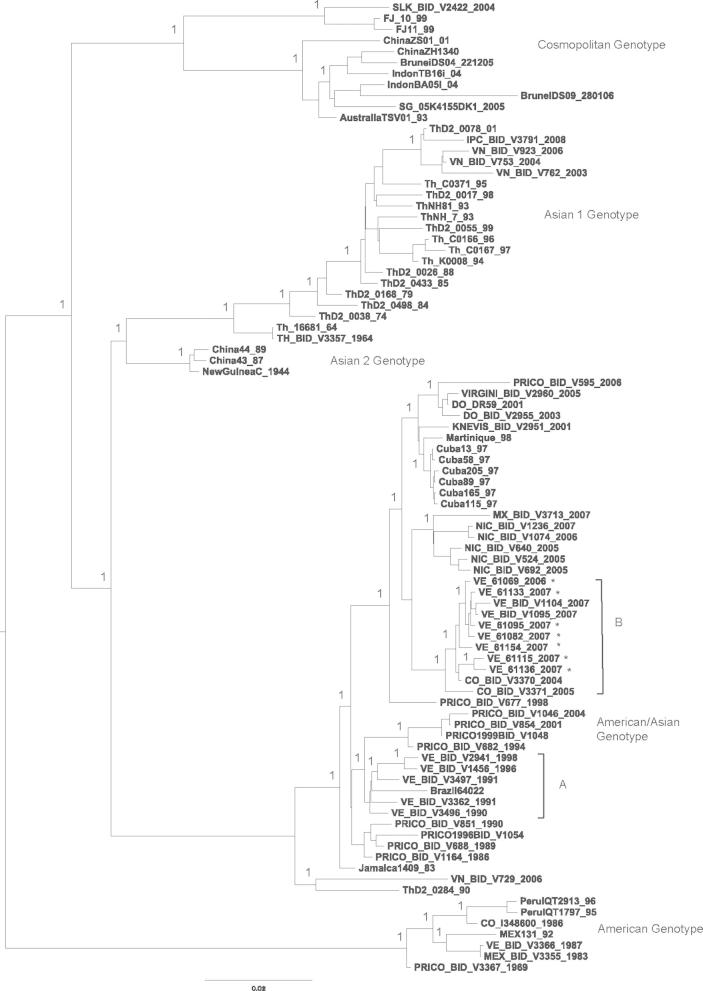
Bayesian phylogeny of the DENV-2 polyprotein nucleotide data set, including Venezuelan isolates from 2006 to 2007. For clarity, posterior probabilities obtained via Bayesian methods are shown for the main clades only. Bootstrap support values obtained via 1000 neighbour-joining replicates were similarly high for these nodes. All horizontal branch lengths are drawn to scale; bar, 0.02 substitutions per site. The tree is midpoint-rooted for purposes of clarity only. The asterisks (∗) indicate those DENV sequences that are published for the first time in the current study.

**Fig. 3:**
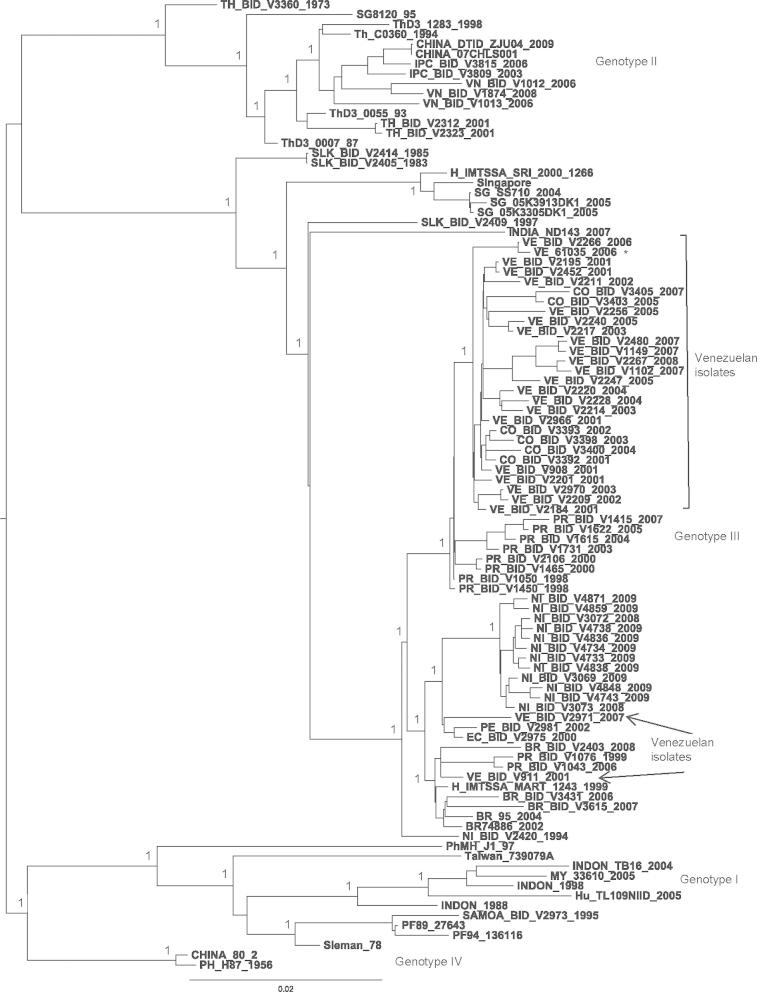
Bayesian phylogeny of the DENV-3 polyprotein nucleotide data set, including Venezuelan isolates from 2006 to 2007. For clarity, posterior probabilities obtained via Bayesian methods are shown for the main clades only. Bootstrap support values obtained via 1000 neighbour-joining replicates were similarly high for these nodes. All horizontal branch lengths are drawn to scale; bar, 0.3 substitutions per site. The tree is midpoint-rooted for purposes of clarity only. The asterisks (∗) indicate those DENV sequences that are published for the first time in the current study.

**Fig. 4:**
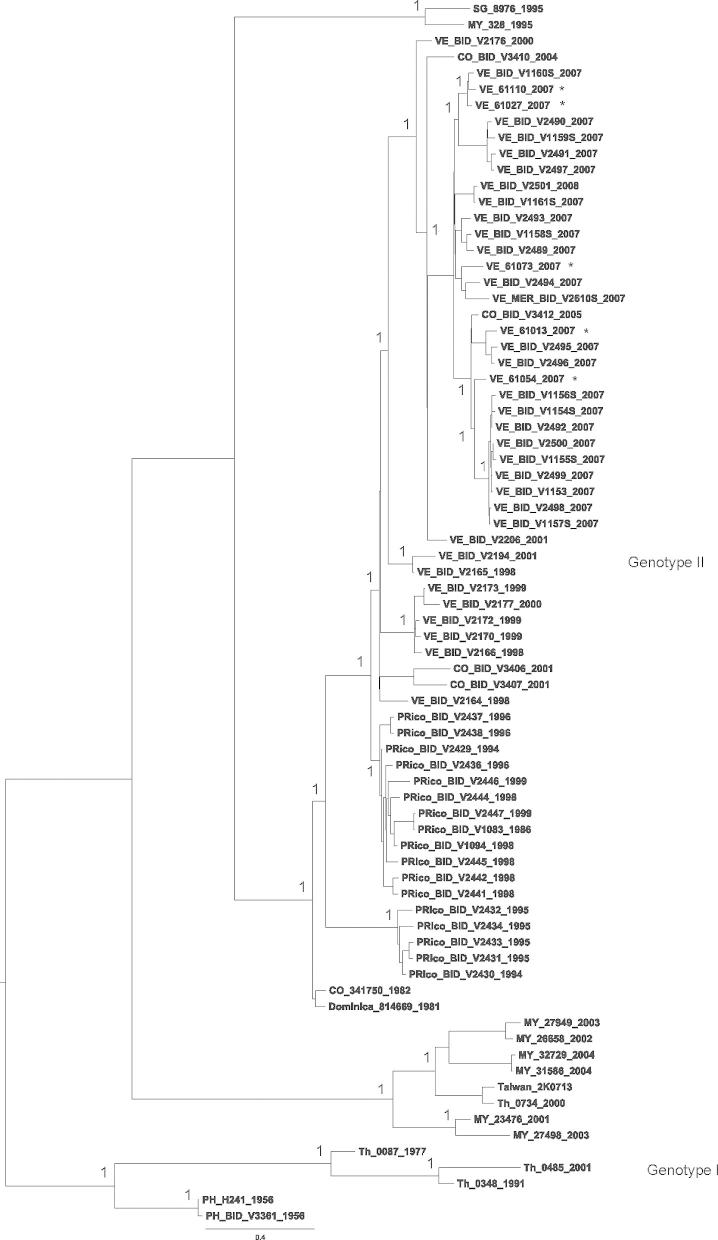
Bayesian phylogeny of the DENV-4 polyprotein nucleotide data set, including Venezuelan isolates from 2006 to 2007. For clarity, posterior probabilities obtained via Bayesian methods are shown for the main clades only. Bootstrap support values obtained via 1000 neighbour-joining replicates were similarly high for these nodes. All horizontal branch lengths are drawn to scale; bar, 0.4 substitutions per site. The tree is midpoint-rooted for purposes of clarity only. The asterisks (∗) indicate those DENV sequences that are published for the first time in the current study.

ML and Bayesian phylogenetic trees were constructed for each dengue serotype. Since robust trees with very similar topologies were obtained by both methods, only Bayesian phylogenies are shown herein (Fig. 1, Fig. 2, Fig. 3, Fig. 4). It should be noted that posterior probabilities obtained via Bayesian methods, and neighbour-joining bootstrap replicate values (not shown), were similarly high for all major nodes.

### 3.1 DENV-1

The Bayesian phylogeny obtained for the DENV-1 data set (Fig. 1) confirms previous results concerning the genetic relatedness of the Venezuelan isolates with other Latin American isolates grouping within a single genotype (Goncalvez et al., 2002). The tree also reveals four sub-clusters within genotype III that include Venezuelan isolates (A–D). However, it is striking that temporal distribution is not observed; isolates from the 1990s are regularly mixed with the most recent isolates. Sub-cluster A is mainly composed of sequences corresponding to recent Venezuelan isolates (2005–2008) including the strain VE_61063_2006 sequenced in this study, one strain from Puerto Rico isolated in 2006 and two Venezuelan isolates from 1997. Sub-cluster B mainly contained Venezuelan isolates (2004–2007) including four isolates sequenced in this study (VE_61059_2006, VE_61060_2006, VE_61068_2006 and VE_61084_2007), which were closely related to Colombian isolates (2005–2007). Sub-cluster C contained two recent Venezuelan isolates (2005 and 2008), a closely related Venezuelan isolate from 1999 and two Colombian isolates from 1998. Finally, sub-cluster D predominantly contained Nicaraguan isolates (2004–2008) but included two Venezuelan isolates from the present study and two Venezuelan isolates from 1997 to 1998. The isolates VE_61006_2006 (from the beginning of November) and VE_61081_2007 (from the end of January, 2007), closely related to strains circulating in Nicaragua (2004–2008), showed 16 amino acid changes in common with the remaining Venezuelan isolates sequenced in the present study i.e. E (1), NS1 (1), NS2A (2), NS2B (5), NS4A (1), NS4B (4) and NS5 (2).

In addition, it is noteworthy that with the exception of the two closely related Nicaraguan isolates, all the Venezuelan isolates corresponding to the period 2006–2007 showed two non-conservative amino acid replacements which appears to be unusual for DENV-1 of any genotype; i.e. D146G (polar acid to polar neutral) in the NS1 protein and H18Y (polar basic to polar neutral) in the NS4B protein. Particularly, the change at NS1 could be of relevance since the motif D prefers generally to be on the surface of proteins and it is quite frequently involved in protein active or binding sites. On the contrary, the motif G can reside in parts of protein structures that are forbidden to all other amino acids. The uniqueness of G also means that it can play a distinct functional role, such as using its side chain-less backbone to bind to phosphates (Betts and Russell, 2003).

### 3.2 DENV-2

The Bayesian tree constructed using the DENV-2 data set (Fig. 2) shows previously identified genotypes (Asian Genotype 1, Asian Genotype 2, Cosmopolitan Genotype, American and American/Asian genotype) (Twiddy et al., 2002). Except for the oldest Venezuelan strain (VE_BID_V3366_1987) included in this analysis, which clustered with the American genotype, all the Venezuelan isolates collected during the 1990s and more recently, clustered within the American–Asian genotype suggesting that the former genotype has been displaced. In general, geographical and temporal distribution in sub-clusters was observed within the American/Asian genotype (e.g. Caribbean isolates were separated from mainland isolates). Interestingly, two major Venezuelan phylogenetic clusters can be distinguished within this genotype. Sub-cluster A corresponds to isolates obtained during the 1990s and sub-cluster B corresponds to Venezuelan isolates collected during the period 2006–2007 which are closely related to Colombian isolates from 2004 to 2005. The isolate CO_BID_3371_2005 is located at the base of this group. Particularly, within sub-cluster B, the Venezuelan isolates VE_61136_2007 and VE_61115_2007 appear slightly separate from the rest of the Venezuelan isolates from 2006 to 2007. These isolates have two non-conservative amino acid replacements, T264I (polar neutral to non-polar neutral) in NS1 protein, and S24P (polar neutral to non-polar neutral) in the region encoding the NS4B protein.

Based on the polyprotein alignment, the more recent Venezuelan isolates differ from earlier isolates collected during the 1990s by twenty amino acid replacements, nine of which appear to be non-conservative and localised in the regions encoding the E (3), NS1 (1), NS2A (2), NS4A (1) and NS5 (2) genes. Three of these nine amino acid replacements, namely I93T (neutral non-polar to neutral polar) in the NS1 protein, A109T (neutral non-polar to neutral polar) and L164R (neutral non-polar to basic polar) both in the NS2A protein, are absent in recent isolates grouped in the American/Asian Genotype with the exception of two strains from Colombia, isolated in 2004 and 2005 which appear closely related to the Venezuelan isolates from 2006 to 2007, suggesting that this lineage may have been circulating earlier. The motif Q164 (neutral polar) in the NS2A protein was conserved amongst DENV-2 of all genotypes except for the Venezuelan strains circulating during the 1990s which contained the motif L (neutral non-polar) at this position and the Venezuelan 2006–2007 and Colombian 2004–2005 isolates which contained R, a basic polar amino acid, which may affect the hydrophobicity of the protein.

### 3.3 DENV-3

The Bayesian phylogeny obtained for the DENV-3 data set (Fig. 3) shows that all Venezuelan isolates group within Genotype III with other Latin American isolates from Nicaragua, Puerto Rico, Brazil, Colombia and Mexico in agreement with previous studies using the envelope gene (Ramirez et al., 2010, Uzcategui et al., 2003). There is a Nicaraguan isolate from 1994 at the base of the group (NI_BID_V2420_1994) coinciding with the year of introduction of this genotype into the Americas (Guzman, 1995, Guzman et al., 1997, Rodriguez-Roche et al., 2005b). Despite the fact that only 2 DENV-3 isolates were recovered from the samples collected in the present study, other Venezuelan sequences published in GenBank that correspond to the period of study, are very close related.

Notably, the Venezuelan cluster includes isolates from 2001 to 2008, mixed only with Colombian isolates from 2002 to 2007, suggesting an extensive virus interchange between Colombia and Venezuela during this period of time. Interestingly, there are two Venezuelan isolates outside this cluster, VE_BID_V911_2001 from Caracas, closely related to Puerto Rican isolates (from 1999 and 2006) and VE_BID_V2971_2007 from Aragua grouping with a Peruvian strain from 2002 and an Ecuadorian strain from 2000, which are located at the base of the cluster that includes the most recent Nicaraguan strains (2008–2009). Although this result suggests that multiple introductions could occur and these lineages could be circulating as non-predominant strains, their transmission has not been demonstrated here as only single isolates were recovered.

Analysis of the DENV-3 polyprotein alignment revealed a unique non-conservative amino acid change in Venezuelan isolates, namely K4E (basic polar to acidic polar) at the N-terminal region of the NS2A protein. Remarkably, this K motif at position 4 is conserved globally amongst DENV-3 but with the exception of strains VE_BID_V911_2001 and VE_BID_V2971_2007 which conserved K, all the Venezuelan isolates from 2001 to 2007 have E at this position. The amino acid K frequently plays an important role in protein structure. Firstly, it can be considered to be amphipathic because the part of the side chain nearest to the backbone is long, carbon containing and hydrophobic, whereas the end of the side chain is positively charged (Betts and Russell, 2003). Therefore the change K4E could be functionally important in the structure of the NS2A protein.

Additional analysis revealed that seven amino acid replacements in the NS5 protein differentiate the Latin American strains from the remaining DENV-3 strains grouped in Genotype III (isolated in Asia) and all the other DENV-3 genotypes. Importantly, one of these changes (S229A) is localised in the core domain of the S-adenosyl methionine-dependent methyltransferase (MTase). The peptide ^223^GNIV**S**SVN^232^ encompassing this change (ie GNIV**A**SVN) is otherwise conserved amongst all four DENV serotypes. However, the change S229A is not observed in the Nicaraguan strain NI_BID_V2420_1994, suggesting that the S229A change occurred *in situ* following its introduction to the Americas in 1994. However, the remaining six changes localised in the RNA-dependent RNA polymerase (RdRp) at positions 365, 371, 374, 389, 422 and 429 were unique to the entire Latin American group in genotype III, including the 1994 Nicaraguan strain.

### 3.4 DENV-4

The Bayesian phylogeny obtained for the DENV-4 data set (Fig. 4) shows that all Venezuelan isolates group within Genotype II with other Latin American isolates. This genotype, known as the Indonesian Genotype (relating to the origin of the virus), was introduced into the Caribbean region in 1981 (Lanciotti et al., 1997). In Fig. 4, the Dominican strain, isolated in 1981, is located at the base of the Latin American group that is mainly represented by Puerto Rican, Colombian and Venezuelan isolates, forming geographic and temporally structured clusters. The Venezuelan isolates corresponding to the present study are closely related to these Venezuelan viruses isolated during the same year and geographical location, as previously published in Genbank. The phylogenetic tree further indicates that in situ evolution of DENV-4 has occurred in Venezuela, with differentiation into a number of distinct but co-circulating lineages, rather than repeated introduction of new strains from other localities. The Venezuelan isolates from Aragua State are much more closely related to the Colombian strains from different periods, as was observed in the DENV-3 phylogeny, confirming significant virus interchanges between these neighbouring countries. The tree also shows dispersal of these lineages within Venezuela, since a sequence retrieved from Genbank corresponding to an isolate from Merida 2007 groups with other 2007 isolates from geographically distant Aragua.

The polyprotein alignment of DENV-4 shows that isolates from Venezuela 2007 and Colombia (2004–2005) have two amino acid replacements in the E protein (A222T and S354A), which are unusual for global DENV-4 strains. According to the complete genome data set these changes were first identified in Venezuela in 2000. Consequently, to examine variation at positions E222 and E354 in more detail, available E gene sequences were retrieved from Genbank. Interestingly, similar to the Venezuelan, Peruvian and Ecuadorian strains isolated in 2000 differ from the strains collected during 2006–2008 at positions E222 and E354. Thus, based on the data in hand the amino acid replacements A222T and S354A appear to have arisen very recently at positions, which are usually invariant. Whether or not these changes impact on viral fitness needs to be determined. However, the replacement at E222 is localised in domain II in a surface exposed loop in the E protein and the replacement at position E354 is localised in the C-terminal domain III (amino acids 303–395) which contains residues associated with changes in host range, tropism, and virulence in different flaviviruses (Rey et al., 1995).

In addition, the 2007 Venezuelan isolates appear to have an amino acid replacement in the C-terminal region of the NS1 protein (D290N). In contrast residue D290 is conserved in all other DENV-4 sequences independent of genotype. Consequently, all isolates grouping within genotype II seem to display D at this position, including the earlier strain Dominica/1981, as well as the Venezuelan isolates collected from 1998 to 2001. Therefore, this analysis suggests that this mutation in the NS1 gene may have been fixed very recently. Although, it is striking that the sylvatic strain MY_P75_215_1975 also has an N residue at NS1 290.

## 4 Discussion

The mechanistic basis for DHF has been a subject of study for decades (Guzman, 2005, Mathew and Rothman, 2008, Tan and Alonso, 2009). Although it is recognised that host and viral factors are the primary determinants for the development of DHF in individual patients, epidemiological and ecological conditions determine the occurrence of DHF epidemics (Guzman and Kouri, 2008). Studies derived from appropriate epidemiological settings have contributed enormously to understanding the underlying basis for the emergence of DHF. Risk factors for the development of severe dengue disease include prior infection with a heterotypic serotype, the strain of the infecting virus, age and gender and the genetic background of the patient (Alvarez et al., 2006, Garcia et al., 2010, Guzman et al., 2002a, Guzman et al., 2000, Kouri et al., 1998, Kouri et al., 1987, Rodriguez-Roche et al., 2011, Sangkawibha et al., 1984, Sierra et al., 2007, Sierra et al., 2006, Vaughn et al., 2000).

Interestingly, on the basis of studies in Venezuela, one particular serotype has predominated during an epidemic period, namely that which replaced the dominant serotype corresponding to the previous period (Salas et al., 1998). Since 1989, all Venezuelan dengue outbreaks have commenced in the central region of the country, particularly in Aragua State, and then spread throughout the entire country (Uzcategui et al., 2003). During the outbreak that occurred from October 1989 to April 1990 which comprised over 6000 DHF cases and 73 deaths (PAHO, 1990), DENV-3 was the only missing serotype and DENV-2 appeared to be associated most frequently with fatal cases (Gubler, 1997, PAHO, 1990). A similar situation occurred from 1997 to 2000 when DENV-2 was once more associated with increasing disease severity (Uzcategui et al., 2001).

During three different epidemics periods (1989, 1997, and 2006) DENV-2 has been associated with increasing disease severity in Venezuela, with the American/Asian genotype being responsible for severe cases. Notably, the phylogenetic tree (Fig. 2) shows that two different DENV-2 lineages have been circulating in Venezuela during the last 20 years both grouped in the American/Asian genotype. One appears to be related to epidemics that occurred at the end of the 1980s and the beginning of the 1990s and subsequently at the end of the 1990s. The other lineage that has been circulating more recently appears to have replaced the previous lineage. Basal to the sub-cluster of recent Venezuelan isolates there are Colombian isolates from 2004 to 2005, suggesting that the second introduction could be from Colombia. Likewise, the Venezuelan isolates show genetic relatedness with earlier isolates from the Caribbean, where increasing severity due to DENV-2 was reported (Guzman et al., 2002b, Rodriguez-Roche et al., 2005a). In the present study, the relatively limited number of samples means that the hypothesis that there is a correlation between disease severity, DENV serotype, sequence of infection and viral genetic differences could not be tested statistically. However, it is notable that according to epidemiological data available all severe cases were only associated with DENV-2; moreover, during the sequencing study three of seven patients infected with this serotype had severe disease. One of these patients (VE_61095_2007) was less than one-year-old and had severe bleeding during primary infection. The other two patients (VE_61115_2007 and VE_61133_2007) both adults, suffered secondary infection in the sequence DENV-3/DENV-2 (Alvarez et al., unpublished results). Because of an absence of bleeding, case VE_61115_2007 was not considered as DHF according to the WHO, 1997 strict classification (WHO, 1997). However, this patient had severe organ impairment and coma; consequently the infection was classified as severe dengue according to the new dengue classification (WHO/TDR, 2009). Case VE_61133_2007, which also had severe organ impairment and coma, was considered as DHF grade II according to the WHO, 1997 strict classification and severe dengue according to the new guidelines TDR/WHO.

No particular viral changes were found that might be directly related with the severe clinical picture observed during the course of DENV-2 infections. For instance, the nucleotide sequence of isolate VE_61095_2007, which caused severe bleeding during dengue primary infection, is very similar to VE_61082_2007 obtained from a patient with non-severe disease. Likewise, isolate VE_61115_2007 (from a severe case) is closely related to VE_61136_2007 (from a non-severe case). Even so, it is important to note that patient VE_61136_2007 showed the highest viral titre among all the studied patients. Consequently, the amino acid changes in the NS2A and NS4B observed only in these two isolates collected late during the epidemic, could account for higher viral fitness. Nevertheless, other non-viral factors could determine the clinical outcome. Recently, it has been shown that a mutation in the NS1 protein of DENV-2 may be associated with intra-epidemic increasing disease severity during secondary infections but presumably this fittest virus can produce asymptomatic and mild disease in the vast majority of primary infected cases (Rodriguez-Roche et al., 2011).

In general, DENV-1 is not frequently associated with a severe disease outcome in the Americas. However, epidemiological data support the possibility that this serotype may play an important role in sensitising the population for a secondary infection (Alvarez et al., 2006, Guzman and Kouri, 2008, Guzman et al., 2002b). Apparently, after the introduction of DENV-1 into Latin America, different lineages evolved independently to generate the diverse genetic variants isolated during the last decade. During the present study it has been demonstrated that different lineages have circulated in Venezuela, presumably introduced from neighbouring countries, where *in situ* evolution of Genotype III has occurred as shown in the DENV-1 tree (Fig. 1). Previous studies using the region encoding the E gene have shown similar results regarding the genetic relatedness of the DENV-1 American isolates (Goncalvez et al., 2002). The extensive genetic variability observed for DENV-1 Venezuelan isolates should be examined considering that the generation of new genetic variants could be linked to increased disease severity. Despite the fact that only one genotype of DENV-1 has been circulating in the Americas since being introduced in 1977, an increasing incidence of severe dengue due to DENV-1 has been reported in Bolivia, 2008 and Argentina, 2009 compared with data corresponding to previous circulation of this serotype during 1999–2000 (Seijo, 2009). Unfortunately, full-length sequences of DENV-1 isolated from these outbreaks are not available.

Similarly, after the introduction DENV-3 (genotype III) in Latin America *in situ* evolution has been observed. Particularly in Venezuela there appears to be a number of distinct but co-circulating lineages, rather than the repeated introduction of new strains from other localities. Only two Venezuelan isolates VE_BID_V911_2001 and VE_BID_V2971_2007 do not fall with the Venezuelan 2000–2008 cluster, indicating that two other genetically distant were introduced although they either have limited circulation or have been under sampled. Recent studies, based on a larger number of envelope gene sequences of DENV-3 genotype III isolated in Venezuela from 2001 to 2008, support this hypothesis since different lineages within genotype III were found, suggesting that several introduction events have occurred into this country. However, the more recent Venezuelan isolates were located in the same cluster as observed in the present study (Ramirez et al., 2010). Nonetheless, it cannot be ignored that several introduced strains from other parts of Latin America may have remained undetected in our study due to sampling bias or gaps in national surveillance. Indeed, in the present study DENV-3 was the least represented amongst the four serotypes. In addition, most sequences included in the analysis (isolates sequenced in this study plus the Venezuelan ones retrieved from Genbank) correspond mainly to Aragua state. DENV-3 may have been displaced in recent years in Aragua state, being the serotype identified the least frequently during the period of the study. Nevertheless, it is important to consider that this serotype could be involved in asymptomatic transmission. For instance, the sequence DENV-2/DENV-3 has been statistically associated with asymptomatic and non-severe disease in other studies (Alvarez et al., 2006). More recently an increase in the circulation of DENV-3 in Venezuela has been reported (Schmidt et al., 2011).

Meanwhile, DENV-4 also shows a pattern of evolution characterised by a number of distinct but co-circulating lineages that do not seem to be associated with repeated introductions. As mentioned above, the pattern observed for DENV-3 and DENV-4 differs from DENV-2 in which replacement of the circulating lineage after a new introduction was associated with increasing proportion of severe dengue cases (Uzcategui et al., 2001).

Most studies concerning the role of the virus in dengue pathogenesis have been focussed on finding viral genetic variants associated with severe disease. However, few studies have examined the evolution of viruses that circulate extensively during hyperendemic transmission without causing severe disease. The results presented here, including the four DENV serotypes, suggest the non-structural proteins could play an important role in DENV evolution, principally NS1, NS2A and NS4B. According to this particular epidemiological setting, changes in these proteins would either be favourable or adverse in terms of viral fitness, since mutations could be associated with severe disease (as possibly occurred with DENV-2) or could be associated with non-severe disease due to the appearance of naturally attenuated strains.

In the present study, the following non-conservative amino acid replacements may be particularly noteworthy: D146G in the NS1 protein and H18Y in the NS4B protein, which are unusual for DENV-1 of any genotype. According to previous reports, changes in NS1 and NS4 proteins could be involved in viral attenuation (Hanley et al., 2003, Kelly et al., 2009). On the contrary, recent studies have demonstrated that mutations in the NS4B protein may increase the efficiency of DENV replication. In addition, it has been suggested that mutations in this protein may also be involved in species tropism of DENV and modulate the balance of efficient replication in mosquito and mammalian cells (Tajima et al., 2011). Moreover, it has been shown that a single amino acid in the non-structural NS4B protein namely L52F confers virulence on DENV-2 in AG129 mice through enhancement of viral RNA synthesis (Grant et al., 2011).

In the present study, the more divergent DENV-2 isolates related to severe dengue cases also showed non-conservative amino acid replacements localised in the NS1 (T264I) and NS4B (S24P) proteins.

The Venezuelan DENV-4 strains isolated during 2007 also showed a particular amino acid change in the NS1 protein at the C-terminal region, which differentiates them from DENV-4 strains of any genotype. Previous studies have demonstrated that the flavivirus NS1 region plays an essential role in the early steps of replication (Lindenbach and Rice, 1997, Mackenzie et al., 1996, Muylaert et al., 1997). Deletion of the C-terminal region of DENV NS1 protein abolishes anti-NS1-mediated platelet dysfunction and bleeding tendency (Chen et al., 2009). Importantly, the C-terminal region of the NS1 protein contains six cysteines, known to play a role in protein folding. The motif NS1 290 that changed in the DENV-4 Venezuelan isolates is located close to the cysteine 8 (position 291) of the NS1 protein (Wallis et al., 2004).

The analysis of the DENV-3 polyprotein alignment revealed that there is a non-conservative amino acid change K4E in the N-terminal region of NS2A protein. This is unusual for DENV-3 of any genotype, but it is specific to the more recent Venezuelan isolates from 2001 to 2007. The genetic variant containing the mutation K4E was detected for the first time in Venezuela in 2001. Although the selective force leading to the selection of this variant has not been identified, it appears to have been fixed because this variant has been circulating in Venezuela for at least 6-years.

The flavivirus NS2A protein is a small (231 amino acids), hydrophobic, multifunctional, membrane-associated protein involved in RNA replication (Chambers et al., 1989, Mackenzie et al., 1998), in host-antiviral interferon response (Liu et al., 2004, Liu et al., 2006, Liu et al., 2005, Munoz-Jordan et al., 2003) and assembly/secretion of virus particles (Kummerer and Rice, 2002, Leung et al., 2008, Liu et al., 2003). In addition, NS2A and also NS4B protein may participate in the modulation of vector competence (McElroy et al., 2006).

A retrospective phylogenetic study of events on the Southern Pacific islands three decades ago, where severe dengue was described in patients infected with the DENV-2 American genotype, recorded attenuation of this virus following a series of outbreaks involving non-synonymous mutations in the NS2A gene (Steel et al., 2010). In contrast, during the latest and most severe DENV-4 epidemic in Puerto Rico in 1998, viruses were distinguished by three amino acid replacements in the NS2A protein (I14V, V54T and P101T), which were fixed far faster than would be expected by drift alone. This study demonstrates viral genetic turnover within a focal population and the potential importance of adaptive evolution in viral epidemic expansion (Bennett et al., 2003).

Finally, the Latin American isolates of DENV-3 genotype III analysed herein showed distinctive amino acid changes in the RNA-dependent RNA polymerase (RdRp). It is known that all pathogenic flaviviruses examined thus far inhibit host interferon responses. In this regard, critical residues in the RdRp domain (355–735) have been defined for the interferon antagonist function of Langat virus NS5 protein (Park et al., 2007).

Long term and short-term studies reveal the association of viral evolution with observed increased severity during epidemics. Despite some advances, the relevance of particular mutations has not been investigated thoroughly. This is in large part due to the lack of suitable animal models with which to study dengue virus “virulence”. Variation in non-structural proteins has been associated with increasing severity in epidemiological settings corresponding to endemic (Bennett et al., 2003; Chen et al., 2008, Klungthong et al., 2004, Tang et al., 2010, Zhao et al., 2010) and non-endemic dengue transmission (Rodriguez-Roche et al., 2005a, Rodriguez-Roche et al., 2005b).

Moreover, evidences indicate that non-structural genetic elements may modulate reduced mosquito competence (Brault et al., 2011).

New episodes of genotype replacement are frequently observed (Vu et al., 2010). However, there are multiple factors implicated in the transmission dynamic of DENVs that remain unclear (reviewed by Holmes, 2009). Epidemiological data have suggested that fitness is always context dependent and that as the immunological landscape changes, viral lineages that evade cross-immunity will be at a selective advantage (Holmes, 2009). In addition, recent studies suggest that strain diversity may limit the efficacy of monoclonal antibody therapy or tetravalent vaccines against DENV as neutralisation potency generally correlated with a narrowed genotype specificity (Brien et al., 2010).

## 5 Conclusions

Collectively, the results presented herein suggest that NS proteins may play an important role in DENV virus evolution, particularly NS1, NS2A and NS4B proteins, although experimental confirmation based on a reverse genetics system are required. The phylogenetic data provide evidence to suggest that multiple introductions of DENV have occurred from the Latin American region into Venezuela and vice versa. The implications of significant viral genetic diversity generated during hyperendemic transmission should be considered with regard to the development and use of tetravalent vaccines. Particular changes in NS proteins could modify viral phenotype, not only in terms of virulence; indeed the identification of changes associated to potential naturally attenuated strains would bring relevant knowledge to the rational design of antivirals and dengue vaccine candidates.

## Competing interests

The authors declare that they have no competing interests.
